# Identification of Quantitative Trait Loci for Grain Quality Traits in a Pamyati Azieva × Paragon Bread Wheat Mapping Population Grown in Kazakhstan

**DOI:** 10.3390/plants14111728

**Published:** 2025-06-05

**Authors:** Akerke Amalova, Simon Griffiths, Aigul Abugalieva, Saule Abugalieva, Yerlan Turuspekov

**Affiliations:** 1 Institute of Plant Biology and Biotechnology, Almaty 050040, Kazakhstan; akerke.amalova@gmail.com (A.A.); absaule@yahoo.com (S.A.); 2 John Innes Centre, Norwich NR4 7UH, UK; simon.griffiths@jic.ac.uk; 3 Kazakh Research Institute of Agriculture and Plant Growing, Almaty Region, Almalybak 040909, Kazakhstan

**Keywords:** *Triticum aestivum* L., quantitative trait loci, mapping population, recombinant inbred lines, grain quality traits

## Abstract

High grain quality is a key target in wheat breeding and is influenced by genetic and environmental factors. This study evaluated 94 recombinant inbred lines (RILs) from a Pamyati Azieva × Paragon (PA × P) mapping population grown in two regions in Kazakhstan to assess the genetic basis of six grain quality traits: the test weight per liter (TWL, g/L), grain protein content (GPC, %), gluten content (GC, %), gluten deformation index in flour (GDI, unit), sedimentation value in a 2% acetic acid solution (SV, mL), and grain starch content (GSC, %). A correlation analysis revealed a trade-off between protein and starch accumulation and an inverse relationship between grain quality and yield components. Additionally, GPC exhibited a negative correlation with yield per square meter (YM2), underscoring the challenge of simultaneously improving grain quality and yield. With the use of the QTL Cartographer statistical package, 71 quantitative trait loci (QTLs) were identified for the six grain quality traits, including 20 QTLs showing stability across multiple environments. Notable stable QTLs were detected for GPC on chromosomes 4A, 5B, 6A, and 7B and for GC on chromosomes 1D and 6A, highlighting their potential for marker-assisted selection (MAS). A major QTL found on chromosome 1D (*QGDI-PA × P.ipbb-1D.1*, LOD 19.4) showed a strong association with gluten deformation index, emphasizing its importance in improving flour quality. A survey of published studies on QTL identification in common wheat suggested the likely novelty of 12 QTLs identified for GDI (five QTLs), TWL (three QTLs), SV, and GSC (two QTLs each). These findings underscore the need for balanced breeding strategies that optimize grain composition while maintaining high productivity. With the use of SNP markers associated with the identified QTLs for grain quality traits, the MAS approach can be implemented in wheat breeding programs.

## 1. Introduction

Wheat (*Triticum aestivum* L.) is a major staple crop worldwide, serving as a primary source of carbohydrate and protein for human consumption [1]. Its widespread use in pasta, bread, and other baked goods underscores its role in global food security. Therefore, enhancing the quality and yield of wheat is critical for meeting increasing market demands and improving human nutrition [2,3]. The grain quality of wheat is controlled by multiple genes and is strongly influenced by environmental factors. Quantitative trait locus (QTL) analysis of bi-parental populations is widely used to uncover the genetic mechanisms underlying these traits and identify markers for marker-assisted selection (MAS). Understanding the genetic and environmental contributions to wheat’s quality is essential for breeding improved cultivars with desirable characteristics.

A range of complex traits important to producers, end-users, and breeders determines wheat’s quality. These include grain characteristics, such as protein content, color, weight, and hardness/texture; milling performance characteristics, including flour yield, protein, moisture, and ash content; flour and dough properties, such as starch content, falling number, gluten quality, and dough rheology; and baking attributes, including the loaf height, volume and texture, dough extensibility, mixing time, cookie diameter, and overall baking score [4].

The genetic regulation of wheat’s grain quality traits is complex, involving multiple QTLs and gene–environment interactions. Recent advances in molecular genetics and high-throughput genotyping have enabled the identification of genomic regions associated with key grain quality traits, yield-related traits, and disease resistance, facilitating the implementation of MAS in breeding programs [5,6,7]. QTL mapping has been instrumental in dissecting the genetic basis of wheat quality, revealing loci linked to protein content, gluten strength, and other important traits [8]. However, despite these advancements, further research is needed to validate the presence of these QTLs across diverse genetic backgrounds and environments, ensuring the reliability and effectiveness of their use in breeding programs.

One of the most critical components of wheat quality is grain protein content (GPC), which directly influences dough properties and end-use suitability. GPC is a quantitative trait controlled by multiple QTLs and strongly influenced by environmental factors [9,10,11,12,13]. Approximately 300 QTLs associated with GPC have been identified across all of wheat’s chromosomes, explaining 0.6% to 66% of phenotypic variance [14]. Among these, *Gpc-B1*, located on chromosome 6BS, is widely used in wheat breeding programs due to its strong association with high protein yield. Despite extensive QTL mapping, the genetic mechanisms regulating GPC remain only partially understood. The only gene that has been found for a high GPC locus is an NAC transcription factor (*NAM-B1*) derived from wild emmer wheat that plays a crucial role in nutrient remobilization [15]. Additionally, *TaNAC019*, an endosperm-specific transcription factor, has been implicated in glutenin and starch accumulation [16]. Understanding the genetic basis of GPC is vital, as higher GPC is preferred for bread and pasta, while lower GPC is desirable for products such as noodles [17].

The presence of gluten proteins is closely linked to GPC and plays a fundamental role in determining the rheological properties of dough [18,19]. Gluten proteins, composed of glutenin and gliadin, contribute to the viscoelasticity of wheat-based products [20,21,22,23]. Glutenin can be further classified into high-molecular-weight glutenin subunits (HMW-GSs) and low-molecular-weight glutenin subunits (LMW-GSs), encoded by the *Glu-1* and *Glu-3* loci located on chromosomes 1A, 1B, and 1D, respectively [24,25,26]. The role of HMW-GSs in determining dough strength has been extensively studied [27,28,29]. In contrast, the contribution of LMW-GSs to grain quality regulation remains less well explored, despite evidence indicating their significant role in dough viscosity and the formation of large gluten polymers [30]. Recent QTL mapping work and GWASs (Genome-Wide Association Studies) have identified major loci for glutenin macropolymer (GMP) content, such as 1DL-2 and 6AS-3, providing valuable markers for wheat breeding programs [31].

Beyond protein content, starch, as the primary storage component of wheat grain endosperm, plays a crucial role in the processing and end-use quality of wheat. Common wheat consists of 25–30% amylose and 70–75% amylopectin, which contributes to grain texture and baking properties [32]. Studies have shown that grain starch content (GSC) is positively correlated with grain size and yield but negatively correlated with grain protein content (GPC), demonstrating the importance of balancing these traits in breeding efforts [33]. Starch synthesis in wheat is regulated by key enzymes, including granule-bound starch synthase I (GBSS I), soluble starch synthase (SSS), branching enzyme (BE), and debranching enzyme (DBE) [34]. However, genetic control of GSC remains largely unexplored beyond the contributions of the well-characterized waxy genes. QTL mapping studies have identified loci for GSC on chromosomes 1A, 1D, 2A, 2D, 4A, 7A, 7B, and 7D [35]. Additionally, a major QTL for B-type granule content has been detected in wild *Aegilops* species [36]. A GWAS, using the 90K SNP assay, further revealed multiple loci associated with granule composition, suggesting that wheat may regulate its starch content through distinct genetic pathways [37].

Developing local mapping populations that accurately reflect regional agricultural conditions is crucial for enabling advanced studies of wheat breeding programs in Kazakhstan. This study investigated the recombinant inbred lines (RILs) of a spring wheat mapping population derived from Pamyati Azieva × Paragon (PA × P) as part of the international ADAPTAWHEAT project [38]. Previous studies using Kompetitive Allele-Specific PCR (KASP) technology have led to the construction of a low-density genetic map for this population [39]. The extensive analysis of the PA × P wheat mapping population has revealed 24 QTLs associated with rust resistance [40,41] and significant variation in yield-related traits in southeastern Kazakhstan [42]. Over a five-year period (2015–2020), further QTL mapping across three different environments in Kazakhstan enabled the identification of 53 stable QTLs for eight agronomic traits out of a total of 296 detected QTLs [43]. The primary purpose of this study was to identify QTLs associated with grain quality traits using data from two contrasting environments in Kazakhstan. By integrating molecular and phenotypic data, this research aimed to enhance breeding strategies and improve wheat quality and yield potential in this region.

## 2. Materials and Methods

### 2.1. Plant Materials and Experimental Design

Previously, we studied a bi-parental mapping population, Pamyati Azieva × Paragon (PA × P), derived from a cross between the spring wheat cultivars Pamyati Azieva (Russia) and Paragon (UK). The PA × P population comprised 94 recombinant inbred lines (RILs) advanced to the F_6_–F_8_ generation through single-seed descent, as described previously [40,41,43]. In Pamyati Azieva, drought and powdery mildew resistance are combined with a high kernel count per spike, ensuring high productivity, and this cultivar is approved for cultivation in northern Kazakhstan and western Siberia. Paragon is a high-quality UK spring wheat with resistance to a broad range of diseases, good straw characteristics, and suitability for both conventional and organic farming [43,44].

The PA × P mapping population, along with the two parents, was planted in experimental plots at the Kazakh Research Institute of Agriculture and Plant Growing (KRIAPG, Almaty, southeastern Kazakhstan) in 2016–2019, and the North Kazakhstan Agricultural Experimental Station (NKAES, Petropavlovsk, northern Kazakhstan) in 2017–2019 (Table S1). Two replications of the mapping population (MP) lines and their parents were planted at each location in completely randomized blocks comprising 1 m^2^ plots. The distance between the rows was 15 cm, with a 5 cm distance between the plants [45]. The weather, climate conditions, and soil types at the two experimental stations have been previously described [43].

### 2.2. Evaluation of Variation in Studied Traits in MP

Phenotypic data were collected for the grain yield and six traits associated with grain quality: test weight per liter (TWL, g/L), grain protein content (GPC, %), gluten content (GC, %), gluten deformation index in flour (GDI, unit), sedimentation value in a 2% acetic acid solution (SV, mL), and grain starch content (GSC, %). All the grain and flour quality analyses were conducted in the grain quality laboratory at the KRIAPG following the GOST (the interstate standard), the registered state technical standards, the standards of a regional standards organization operating under the auspices of the Commonwealth of Independent States and maintained by the Euro-Asian Council for Standardization, Metrology, and Certification, and the guidelines of the American Association of Cereal Chemists (AACC) and the International Organization for Standardization (ISO) [46,47]. The specific state and international standards applied in this study are listed in Table 1.

The TWL was determined in g/L according to GOST 10840-2017 “Grain. Method for determination of hectolitre weight” [48]. The GPC was measured by the Kjeldahl method using near-infrared spectroscopy (NIRS DS2500 Grain Analyzer FOSS, Hillerød, Denmark). The GSC and GC were measured using the NIRS DS2500 Grain Analyzer (FOSS, Hillerød, Denmark), with calibration provided by the manufacturer, and the level of flour sedimentation was determined using a 2% acetic acid solution. According to the established classifications, the wheat was categorized as weak (0–30 mL), a filler (31–50 mL), valuable (51–70 mL), or strong (>70 mL) [55]. GDI was evaluated using the IDK-1 apparatus (CJS Company “Prompribor”, Kursk, Russia).

Grain quality traits were interpreted according to the State Standard (GOST) [56]. This classification is based on a range of key grain quality parameters, including GPC, GC, GDI, TWL, falling number, vitreousness, moisture content, and the presence of weed and grain impurities. Table 2 presents an excerpt from the standard, focusing only on the traits relevant to this study.

All classifications and measurements were conducted according to the guidelines specified in the national wheat quality standards.

Additionally, the thousand kernel weight (TKW) and yield per square meter (YM2) of the mapping population (MP) were analyzed to assess the relationships between grain quality and yield-related traits. The YM2 was measured for a 1 m^2^ plot and TKW by weighing 1000 seeds.

### 2.3. Genotyping of the Mapping Population, QTL Analysis, and Statistics

The 94 RILs and two parental cultivars were genotyped using Illumina’s iSelect 20K single nucleotide polymorphism (SNP) array by TraitGenetics (TraitGenetics GmbH, Gatersleben, Germany). The genotypic data were filtered to exclude markers with more than 10% missing data and a minor allele frequency below 0.1, resulting in 4595 polymorphic SNP markers. QTL identification was conducted using the composite interval mapping (CIM) method in Windows QTL Cartographer version 2.5 (LOD ≥ 3.0) [57]. For each detected QTL, the additive effect was calculated as the average effect of substituting one allele for another. The phenotypic variation (R^2^) explained by each QTL was also determined, indicating the percentage of total phenotypic variance attributable to that locus. Both values were extracted from the CIM output generated using WinQTL Cartographer. The QTLs were classified based on the proportion of the phenotypic variation they explained (R^2^ value): major QTLs accounted for >10%, while minor QTLs accounted for <10% [58]. Stable QTLs were those detected in two or more conditions (environment/location or over the years). A genetic map was drawn using MapChart version 2.32 software [59], and significant SNPs were analyzed to identify any genes overlapping with the Wheat Chinese Spring IWGSC RefSeq version 1.0 genome using Ensembl Plants BLAST (https://plants.ensembl.org/Triticum_aestivum/Tools/Blast, accessed on 6 October 2024) [60]. Pearson’s correlation and boxplot analyses were performed using the R statistical platform version 4.3.0 (POSIT, Boston, MA, USA) [61].

## 3. Results

### 3.1. Phenotypic Variations of Quality Traits

**Figure 1:**
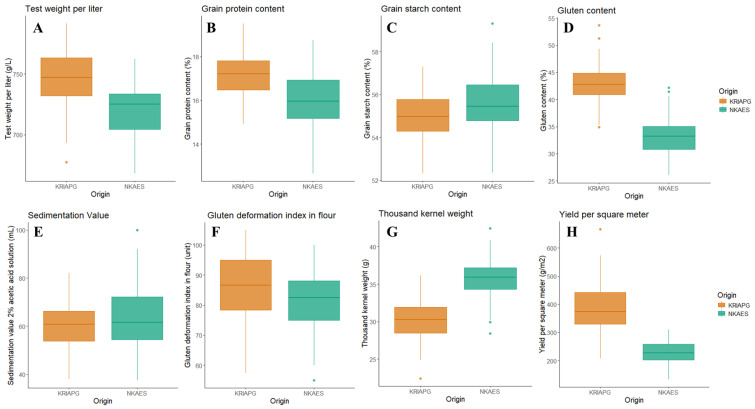
The distribution of the grain quality and yield-related traits of recombinant inbred lines of the PA × P population grown in two regions in the north (NKAES) and southeast (KRIAPG). Note: test weight per liter (**A**), grain protein content (**B**), grain starch content (**C**), gluten content (**D**), gluten deformation index (**E**), sedimentation value (**F**), thousand kernel weight (**G**), and yield per square meter (**H**).

Six grain quality traits were evaluated in ninety-four recombinant inbred lines (RILs) of the PA × P mapping population from the southeast (KRIAPG) and north (NKAES) of the country (Figure 1), along with yield component traits such as the TKW and YM2. The analysis revealed significant variations in grain quality traits across the two locations (KRIAPG and NKAES). The TWL ranged from 718.6 ± 2.18 g/L at the NKAES to 745.8 ± 2.04 g/L at the KRIAPG. The mean GPC was higher at the KRIAPG (17.2 ± 0.10%) than at the NKAES (16.1 ± 0.13%), and a similar trend was observed for GC, which was 10% higher at the KRIAPG than at the NKAES. In contrast, GSC was 8% higher at the NKAES (63.8 ± 1.34%) than at the KRIAPG (55.0 ± 0.11%). Furthermore, the mean SV was higher at the NKAES (63.1 ± 1.34 mL) than at the KRIAPG (60.3 ± 1.03 mL). The values of this indicator in the parental varieties were higher in the southeast than in the north (Figure 1). In general, *t*-tests suggested that the average values of TWL, GPC, GSC, GC, GDI, and SV in the two contrasted regions were significantly different (*p* < 0.0001) (Table S1).

**Figure 2:**
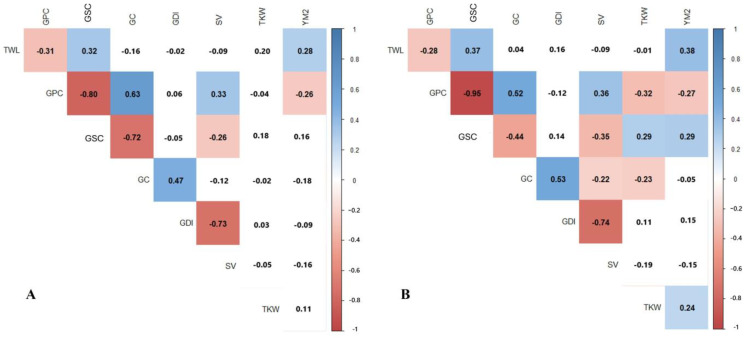
Pearson’s correlation index for averaged data of eight traits associated with yield and grain quality in 94 RILs of PA × P population grown in the north (NKAES) (**A**) and southeast (KRIAPG) (**B**) of Kazakhstan. Note: TWL—test weight per liter (g/L); GPC—grain protein content (%); GC—gluten content (%); GDI—gluten deformation index (unit); SV—sedimentation value (mL); GSC—grain starch content (%); TKW—thousand kernel weight (g); YM2—yield per square meter (g/m^2^). Correlations with *p* < 0.05 are highlighted in blue (positive) or red (negative).

The correlation analysis revealed a strong negative relationship between GPC and GSC, with correlation coefficients of −0.80 at the NKAES and −0.95 at the KRIAPG. A negative correlation was also observed between GPC and YM2 at both sites. GDI showed a consistent negative correlation with SV, with coefficients of −0.73 in the NKAES and −0.74 at the KRIAPG. Additionally, positive correlations were found between GC and both GSC and GPC, ranging from 0.47 to 0.53 (Figure 2).

Five grain quality traits were assessed in a total of 94 RILs following the state standards of the Republic of Kazakhstan for bread wheat classification (Table 2). For GPC, the majority of RILs at both locations met the first class standard (14.5–19.0%), including 88 at the NKAES and all 94 at the KRIAPG. A small number of lines fell into the second (three RILs) and third (one RIL) classes at the NKAES, with two RILs remaining unclassified (Table 3).

The assessment of the GC showed that all of the RILs at the KRIAPG met the first-class standard (≥32%), while at the NKAES, 58 RILs were classified as first class, 27 as second class (≥28%), and five as third class (≥23%). Four RILs were unclassified. The analysis of GDI categorized the RILs into Group I (optimal range: 43–77 units) and Group II (broader range: 18–102 units). At the NKAES, 32 RILs belonged to Group I and 62 to Group II; 4 were unclassified. At the KRIAPG, 22 RILs were in Group I, 72 were in Group II, and 4 were unclassified (Table 3).

SV, which reflects gluten strength, varied across the regions. At the NKAES, 28 RILs were classified as “strong” (>70 mL), 46 as “valuable” (51–70 mL), and 17 as being of “filler” quality (31–50 mL). At the KRIAPG, 17 RILs were “strong,” 58 “valuable,” and 19 “filler”. Three RILs at the NKAES were unclassified (Table 3).

Regarding TWL, an important physical grain quality trait, 38 RILs from the KRIAPG and 44 from the NKAES were assigned to the first/second class category (≥750 g/L). In contrast, 35 and 27 RILs were classified as third class (≥730 g/L) in KRIAPG and NKAES, respectively. Furthermore, 19 RILs (KRIAPG) and 30 RILs (NKAES) were assigned to the fourth class (≥710 g/L). Notably, 33 RILs at the NKAES and 2 at the KRIAPG remained unclassified for the TWL. Four RILs (PA × P-03, PA × P-14, PA × P-20, and PA × P-28) were consistently classified as first or second class in both environments. In terms of GSC, all samples demonstrated values below the required standard range of 65–70% (Table S1).

Eight RILs (PA × P-86, PA × P-25, PA × P-14, PA × P-03, PA × P-12, PA × P-92, PA × P-38, and PA × P-19) belonged to the highest performance classes for four grain-related traits in southeastern Kazakhstan. Six RILs (PA × P-73, PA × P-05, PA × P-20, PA × P-14, PA × P-03, and PA × P-28) similarly belonged to the highest performance classes based on several grain-related traits under the conditions of northern Kazakhstan. Notably, two lines, PA × P-14 and PA × P-03, consistently demonstrated superior performance across both regions. Overall, more of the RILs evaluated in the KRIAPG met higher quality standards across the traits.

### 3.2. QTL Mapping of Quality Traits in Northern and Southeastern Regions

A QTL analysis of six grain quality traits was conducted on 94 RILs from the PA × P mapping population at two locations (the NKAES, northern Kazakhstan, and the KRIAPG, southeastern Kazakhstan) during the 2016–2019 harvest seasons (Table S2 and Table 4). A total of 71 QTLs were identified, with 60 classified as major (R^2^ of >10%) (Table S2). Additionally, 20 stable QTLs were detected under two or more conditions (environment/location or over the years). The number of stable QTLs for the analyzed quality indicators ranged from two (for GSC) to five (for GDI) (Table S2 and Table 4).

A high LOD value (19.4) was found for the *QGDI-PA × P.ipbb-1D.1* locus on chromosome 1D, identified by the GDI in both the northern and southeastern regions. The number of stable QTLs was ten for the A genome and five each for the B and D genomes. In total, fourteen stable QTLs were detected for six quality traits across both regions.

For TWL, 15 QTLs were mapped to 11 chromosomes (Table S2). Among them, *QTwl-PA × P.ipbb-1A*, *QTwl-PA × P.ipbb-4A*, and *QTwl-PA × P.ipbb-7D* were classified as stable and were localized on chromosomes 1A, 4A, and 7D, respectively (Table 5).

**Figure 3:**
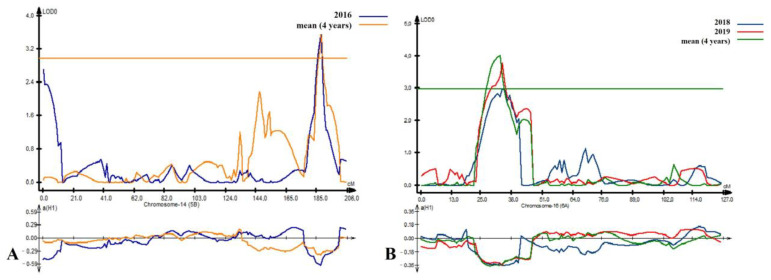
Position of identified quantitative trait loci (QTLs) for grain protein content (GPC), on chromosomes 5B (**A**) and 6A (**B**).

For GPC, twelve QTLs were detected on nine chromosomes: 3A, 3B (2 QTLs), 4A, 5A, 5B, 6A (2 QTLs), 6B, 7A (2 QTLs), and 7B (Table S2). The LOD values ranged from 3.1 to 6.0, and the proportion of the phenotypic variation (R^2^) explained by the 11 major QTLs varied from 10% to 40% (Table S2). Only four QTLs (*QGpc-PA × P.ipbb-4A*, *QGpc-PA × P.ipbb-5B*, *QGpc-PA × P.ipbb-6A*, and *QGpc-PA × P.ipbb-7B*) were stable. *QGpc-PA × P.ipbb-4A* and *QGpc-PA × P.ipbb-7B* were detected at the NKAES (2018–2019), with additive effects of 0.39 (Pamyati Azieva) and 0.47 (Paragon), respectively. *QGpc-PA × P.ipbb-5B* and *QGpc-PA × P.ipbb-6A* were found at the KRIAPG (2016, 2018, 2019) with additive effects of 0.36 and 0.35 (both from Paragon) (Table S3 and Table 5, Figure 3).

**Figure 4:**
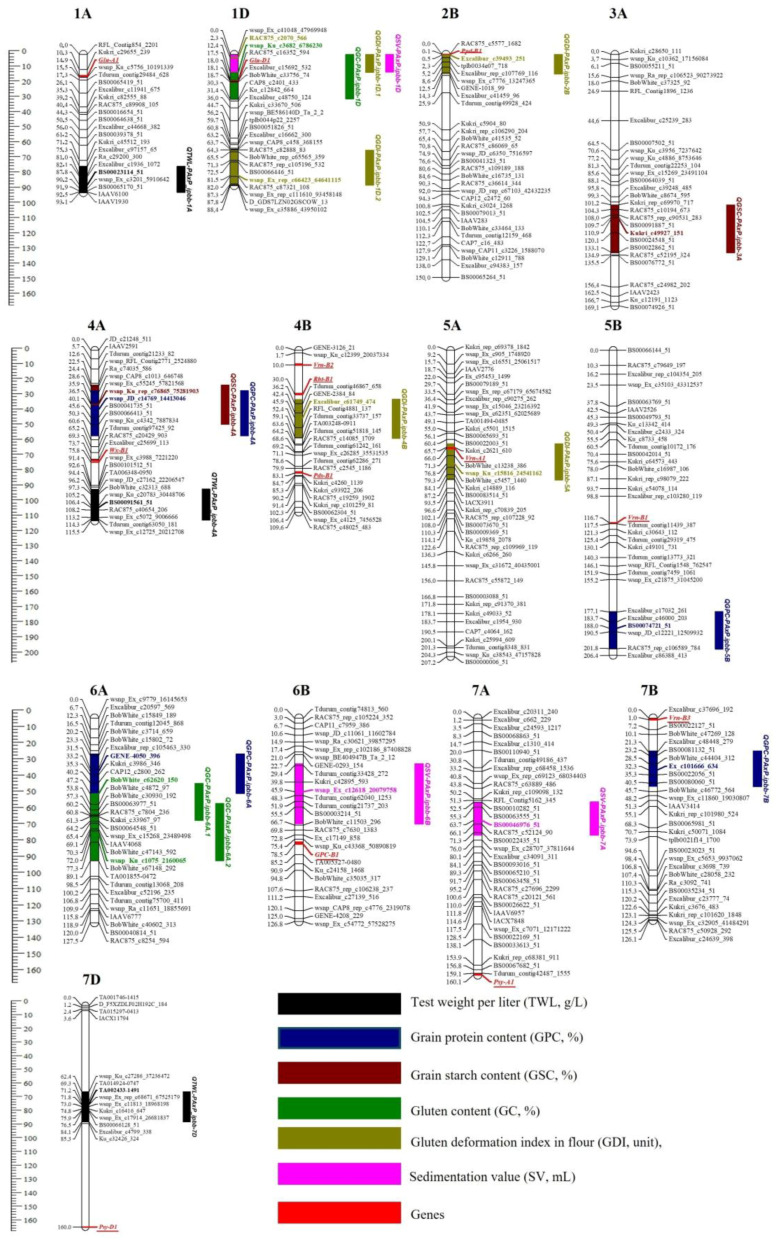
Genetic map of Pamyati Azieva × Paragon mapping population showing QTLs associated with grain quality traits.

For the GC, 11 QTLs were identified on chromosomes 1D (3 QTLs), 2B, 3A, 3B, 5A, 6A (three QTLs), and 6B (Table S2). Among them, only three QTLs (*QGC-PA × P.ipbb-1D*, *QGC-PA × P.ipbb-6A.1*, and *QGC-PA × P.ipbb-6A.2*) were stable. The *QGC-PA × P.ipbb-1D* locus was identified in both regions, with LOD values ranging from 3.5 to 9.7 and an additive effect of −2.45% when the Paragon allele was present (Table S3 and Table 5, Figure 4).

For the GDI, a total of 11 QTLs were identified on chromosomes 1D (3 QTLs), 2B, 3D, 4A, 4B, 5A (3 QTLs), and 5B (Table S2). However, only five QTLs were classified as stable and were detected in multiple environments. The *QGDI-PA × P.ipbb-1D.1* locus showed an LOD range of 13.7 to 19.4 and an additive effect of −8.48 units from the Paragon allele, while the *QGDI-PA × P.ipbb-1D.2* locus had an LOD of 3.6 to 9.2, with an additive effect of 4.76 units inherited from Pamyati Azieva (Table S3 and Table 5, Figure 4).

For the SV, eight major QTLs were detected on five chromosomes: 1D (two QTLs), 2B, 5B, 6B (2), and 7A (two QTLs) (Table S2). The loci *QSed-PA × P.ipbb-1D*, *QSed-PA × P.ipbb-6B*, and *QSed-PA × P.ipbb-7A* were identified under two or more different conditions, confirming their stability. The *QSed-PA × P.ipbb-1D* locus was detected in both regions from 2016 to 2019, with LOD values ranging from 6.7 to 16.9 and an additive effect of 14% when the Pamyati Azieva allele was present (Table S3 and Table 4, Figure 4).

Twelve major QTLs for the GSC were identified on eight wheat chromosomes: 1D, 3A, 3B (2 QTL), 4A, 4B, 6A (2 QTLs), 7A (2 QTLs), and 7B (2 QTLs) (Table S2). The LOD values of the detected QTLs varied from 3.4 to 4.8, with phenotypic variance (R^2^) ranging from 11% to 19%. Two QTLs (*QGsc-PA × P.ipbb-3A* and *QGsc-PA × P.ipbb-4A*) were classified as stable and the Paragon allele had an additive effect on those QTLs identified in both environments (Table S3 and Table 4, Figure 4).

The QTL analysis demonstrated strong genetic control of grain quality traits, with the identification of major and stable QTLs being useful for MAS. In particular, chromosome 1D appeared to be significant for multiple traits (GDI, GC, and SV), making it a target for further fine mapping and functional validation.

## 4. Discussion

### 4.1. Phenotypic Variability of and Environmental Influence on 94 RILs in the Pamyati Azieva × Paragon Mapping Population

The evaluation of the PA × P mapping population conducted in two distinct environments in northern Kazakhstan (NKAES) and southeastern Kazakhstan (KRIAPG) revealed significant variability in the grain quality traits. Traits such as GPC, GC, SV, and GSC were subject to strong environmental influence, reflecting the complex interplay between genetic factors and regional climatic conditions [12]. Notably, GPC and GC were higher at the KRIAPG, while GSC and SV were higher at the NKAES. These findings align with the results of previous studies, which demonstrate the sensitivity of wheat quality traits to environmental factors, particularly temperature and soil conditions [42,43]. Significant negative correlations were observed between GPC and GSC (−0.80 at the NKAES, −0.95 at the KRIAPG), confirming the trade-off between protein and starch accumulation [33,62]. Additionally, GPC exhibited a negative correlation with YM2, underscoring the difficulty in simultaneously improving grain quality and yield [63]. These results highlight the importance of balanced breeding strategies that optimize grain composition while maintaining high productivity [19,64]. The positive correlation between GC and GPC suggests that selecting higher protein content could also improve gluten strength, which is desirable for bread-making [19,65,66].

### 4.2. Identification and Stability of QTLs for Grain Quality Traits

The present study conducted across two different regions of Kazakhstan over four years identified 71 QTLs associated with six grain quality traits in the PA × P mapping population. Among these, 60 QTLs located on 19 chromosomes were classified as major QTLs (R^2^ > 10%), and 20 showed stability across multiple environments or over the years (Table 4 and Table 5). The analysis of the genomic distribution of these stable QTLs revealed that the A genome contained the highest number (10), followed by the B and D genomes (5). Notably, 15 out of the 20 stable QTLs being identified in both study regions suggests that these loci exert a powerful influence under various environmental conditions, making them valuable targets for marker-assisted selection. The 12 novel QTLs identified in this study were associated with TWL (three QTLs), GSC (two QTLs), GDI (five QTLs), and SV (two QTLs), presenting valuable opportunities for MAS, which enables the development of wheat varieties with superior quality and yield potential.

A comparison of our QTL mapping results with QTLs mapped in previously published studies showed that eight of the twenty QTLs were co-localized in the same genetic position as those found in earlier studies (Table S4). Most of these overlaps were observed for QTLs related to GPC (four QTLs), followed by those related to GC (three QTLs) and SV (one QTL). For example, *QGpc-PA × P.ipbb-4A* and *QGpc-PA × P.ipbb-7B* were detected in tests performed in northern Kazakhstan, whereas *QGpc-PA × P.ipbb-5B* and *QGpc-PA × P.ipbb-6A* were found in experiments conducted in the southeastern region, highlighting the influence of environmental factors on the distribution of these strains. A literature review confirmed that these QTLs had been previously reported, supporting the validity of our findings [11,67,68,69] (Table S4). Among the QTLs for GC, *QGC-PA × P.ipbb-1D* was detected in both locations and exhibited a significant additive effect, indicating its potential role in enhancing gluten strength. Our results aligned with those of previous studies [70,71], which identified QTLs for GC (*QGC-PA × P.ipbb-1D*, *QGC-PA × P.ipbb-6A.1*, and *QGC-PA × P.ipbb-6A.2*) in similar genetic positions.

Similarly, SV, an important indicator of gluten quality, was associated with the QTL *QSed-PA × P.ipbb-1D* detected on chromosome 1D in six environments, consistent with previous findings on grain quality traits in Indian wheat germplasm [72]. Furthermore, *QGDI-PA × P.ipbb-1D.1*, which showed the highest LOD value (19.4), was identified in both locations, highlighting its major role in determining gluten extensibility. Similarly, two QTLs (*QGDI-PA × P.ipbb-2B* and *QGDI-PA × P.ipbb-5A*) associated with GDI were located on chromosomes 2B and 5A, aligning with the positions of *Ppd-B1* and *Vrn-A1*, at 0.0 cM and 66.0 cM, respectively, in the Wheat-Composite2004 map available in the GrainGenes database [73]. Notably, *Ppd-B1* is involved in photoperiod sensitivity, and *Vrn-A1* regulates vernalization response, with both genes influencing flowering time and, consequently, grain development [74,75]. Their co-localization with QTLs for gluten deformation suggests a potential pleiotropic effect or close linkage between developmental genes and gluten quality traits, reinforcing the complex interplay between phenology and end-use quality in wheat. QTLs related to GC, SV, and GDI were co-located on chromosome 1D (0.0–30.0 cM), suggesting a pleiotropic effect. A literature review revealed that these QTLs were situated near the well-known *Glu-D1* locus found at 18.7 cM on the Triticum-Genes-1D map available in the GrainGenes database [73]. The *Glu-D1* locus encodes high-molecular-weight glutenin subunits, which are critical determinants of dough strength and elasticity, underscoring the importance of this genomic region in controlling gluten quality traits [23].

Interestingly, pleiotropic loci were identified in this population, highlighting genetic relationships between grain quality traits, plant adaptation, and yield-related traits [43]. Three pleiotropic loci were detected for adaptability traits and yield components, including QTLs for thousand-kernel weight and TWL on chromosome 1A (74.3–93.0 cM). Additionally, pleiotropic loci were identified for spike length, GPC, and GSC on chromosome 4A (26.6–45.3 cM). Furthermore, a locus on chromosome 7B (20.4–42.7 cM) was found to influence heading time and GPC simultaneously, suggesting a genetic link between phenology and grain nutritional quality (Table S5).

The identification of stable and major QTLs for GPC, GC, SV, and GDI across multiple environments in this study offers promising tools for MAS in wheat breeding programs. The QTLs that showed consistent effects across both study regions are particularly valuable for developing cultivars with improved grain quality traits under diverse agro-climatic conditions. Moreover, the co-localization of some quality QTLs with *Ppd-B1*, *Vrn-A1*, and *Glu-D1* highlights opportunities for pyramiding favorable alleles that simultaneously enhance yield components, phenology, and grain quality traits. Additionally, the development of KASP markers based on SNPs tightly linked to stable QTLs will facilitate high-throughput genotyping and cost-effective selection of desirable alleles in early-generation breeding populations. Overall, the validated markers linked to quality traits identified in this study represent valuable molecular tools for the accelerated development of wheat varieties with improved grain quality and broad environmental adaptation.

### 4.3. Functional Annotation and Potential Candidate Genes

Significant SNPs associated with the 20 stable QTLs were aligned with the Chinese Spring reference genome [76], using the Wheat Ensembl database [60] to further elucidate the functional relevance of these loci. The results revealed that thirteen QTLs were located in genic regions, whereas seven were in intergenic positions (Table S3). Among the twelve novel QTLs identified, eight SNPs were mapped to genic regions, suggesting their functional significance in regulating grain quality traits. The functional annotation of the SNPs linked to these QTLs identified candidate genes involved in protein biosynthesis, lipid metabolism, enzyme activity, nitrogen metabolism, and transcriptional regulation. Notably, genes encoding transcription factors such as NB-ARC domain-containing protein were enriched in the identified QTL regions, highlighting their potential roles in regulating grain yield in rice [77]. Zinc finger B-box proteins and NB-ARC proteins are likely candidates for providing resistance to stress from drought, light, and salinity [78]. Another protein, ABC transporter B family protein, may contribute to detoxification and stress tolerance mechanisms [79]. These findings provide valuable insights into the genetic control of wheat’s grain composition and offer promising targets for future breeding programs to enhance grain quality and facilitate adaptation to diverse environments.

## 5. Conclusions

The present study evaluated six grain quality traits in 94 RILs of the PA × P mapping population across two contrasting regions in Kazakhstan. A significant environmental influence on GPC, GC, SV, and GSC was observed, highlighting the interaction between genetic factors and climatic conditions. A correlation analysis confirmed a negative association between the protein and starch content, with the same relationship noted between grain quality traits and yield components. A QTL analysis identified 71 QTLs associated with six grain quality traits, with 20 QTLs showing stability across multiple environments. Specifically, four stable QTLs for GPC were detected on chromosomes 4A, 5B, 6A, and 7B, whereas three stable QTLs for GC were found on chromosomes 1D and 6A. A survey of published studies on common wheat QTL identification suggested that twelve of the QTLs for the six analyzed traits may have been novel genetic factors, while eight QTLs matched associations found in previous research. The detection of 15 QTLs in both the northern and southeastern regions, including those for GPC, GC, GSC, and SV, emphasizes their robustness under different environmental conditions. The stable QTLs identified can be effectively utilized in MAS to develop wheat varieties with improved grain quality and adaptability to diverse environmental conditions.

## Figures and Tables

**Table 1:** Standards for determining grain quality traits.

Grain Quality Trait	Standards	
	Interstate	International
Test weight per liter (TWL, g/L)	GOST 10840-2017 [48]	AACC 55-10 [46]
Grain protein content (GPC, %)	GOST 10846-91 [49]	ISO 1871:2009 [47,50]
Grain starch content (GSC, %)	GOST 10845-98 [51]	-
Gluten content (GC, %)	GOST 13586.1-68 [52]	ISO 21415-1:2006 [47,53], ISO 21415-2:2006 [47,54]

**Table 2:** Interstate standard requirements for determining the quality of bread wheat grain [56].

Name of the Traits	Characteristics of Bread Wheat by Classes				
Class	1st	2nd	3rd	4th	5th
Grain protein content, %	≥14.5	≥13.5	≥12.0	≥10.0	Not limited
Gluten content, %	≥32.0	≥28.0	≥23.0	≥18.0	Not limited
Gluten deformation index, unit	I group		II group		Not limited
	43–≤77		18–≤102		
Test weight per liter, g/L	≥750		≥730	≥710	Not limited

**Table 3:** Characterization of samples in the PA × P mapping population using five grain quality traits.

Grain protein content (GPC, %)		
Classes	NKAES	KRIAPG
1 class (14,5–19.0%)	88 RILs	94 RILs
2 class (13.5%)	3 RILs	-
3 class (12.0%)	1 RILs	-
Unclassified	2 RILs	-
Gluten content (GC, %)		
Classes	NKAES	KRIAPG
1 class (32%)	58 RILs	94 RILs
2 class (28%)	27 RILs	-
3 class (23%)	5 RILs	-
Unclassified	4 RILs	-
Sedimentation value (SV, mL)		
Classes	NKAES	KRIAPG
Strong (>70 mL)	28 RILs	17 RILs
Valuable (51–70 mL)	46 RILs	58 RILs
Filler (31–50 mL)	17 RILs	19 RILs
Unclassified	3 RILs	-
Gluten deformation index (GDI, unit)		
Group	NKAES	KRIAPG
I (43–77 unit)	30 RILs	22 RILs
II (18–102 unit)	60 RILs	68 RILs
Unclassified	4 RILs	4 RILs
Test weight per liter (TWL, g/L)		
Classes	NKAES	KRIAPG
1/2 class (750–790 g/L)	4 RILs	38 RILs
3 class (730 g/l)	27 RILs	35 RILs
4 class (710 g/l)	30 RILs	19 RILs
Unclassified	33 RILs	2 RILs

**Table 4:** Number of identified quantitative trait loci in Pamyati Azieva × Paragon wheat mapping population in two study locations.

Traits	All QTL	Major QTL ^1^	Stable QTL ^2^	Stable QTL	
				NKAES	KRIAPG
Test weight per liter (TWL, g/L),	15	15	3	3	3
Grain protein content (GPC, %)	12	11	4	2	2
Gluten content (GC, %),	11	11	3	2	3
Gluten deformation index in flour (GDI, unit)	11	2	5	5	5
Grain starch content (GSC, %)	12	12	2	2	2
Sedimentation value (SV, mL)	10	9	3	2	3
Total	71	60	20	16	18

Note: 1—major QTLs (R^2^ > 10%), 2—stable QTLs detected in two or more environments.

**Table 5:** List of identified QTLs for grain quality traits of the mapping population Pamyati Azieva × Paragon in two regions of Kazakhstan (harvest from 2016 to 2019).

Traits	QTL	Chromosome	Interval cM	LOD	Max. R^2^ %	Additive		Conditions (Region, Year)
						Effect	Allele	
TWL	*Qtwl-PA × P_ipbb- 1A*	1A	75.2–93.0	4.2	12	12.2	Pamyati Azieva	KRIAPG-19, NKAES-18
TWL	*Qtwl-PA × P_ipbb-4A*	4A	94.0–115.0	4.8	17	10.5	Pamyati Azieva	KRIAPG-17, NKAES-17, NKAES 3-years mean
TWL	*Qtwl-PA × P_ipbb-7D*	7D	62.4–84.2	4.7	34	12	Pamyati Azieva	KRIAPG-18, KRIAPG 4-years mean, NKAES-19, NKAES 3-years mean
GPC	*QGpc-PA × P.ipbb-4A*	4A	27.2–58.0	6	24	0.39	Pamyati Azieva	NKAES-19, NKAES 3-years mean
GPC	*QGpc-PA × P.ipbb-5B*	5B	176.7–202.2	3.9	12	−0.36	Paragon	KRIAPG-16, KRIAPG 4-years mean
GPC	*QGpc-PA × P.ipbb-6A*	6A	22.3–47.1	4	13	−0.35	Paragon	KRIAPG-18, KRIAPG-19, KRIAPG 4-years mean
GPC	*QGpc-PA × P.ipbb-7B*	7B	20.4–42.7	4	12	−0.47	Paragon	NKAES-18, NKAES 3-years mean
GSC	*QGsc-PA × P.ipbb-3A*	3A	101.2–133.6	4	15	−0.51	Paragon	KRIAPG-18, KRIAPG 4-years mean, NKAES-18
GSC	*QGsc-PA × P.ipbb-4A*	4A	23.4–50.2	5.4	19	−0.46	Paragon	KRIAPG 4-years mean, NKAES-19, NKAES 3-years mean
GC	*QGC-PA × P.ipbb-1D*	1D	0.0–30.0	9.7	32	−2.47	Paragon	KRIAPG-18, NKAES-17, NKAES-19, NKAES 3-years mean
GC	*QGC-PA × P.ipbb-6A.2*	6A	40.6–63.8	4.9	17	−1.41	Paragon	KRIAPG-19, KRIAPG 4-years mean
GC	*QGC-PA × P.ipbb-6A.1*	6A	53.2–88.9	6.5	20	−1.86	Paragon	KRIAPG-18, NKAES-18
GDI	*QGDI-PA × P.ipbb-1D.1*	1D	0.0–12.3	19.4	53	−8.48	Paragon	KRIAPG-17, KRIAPG-18, KRIAPG-19, KRIAPG 4-years mean; NKAES-17, NKAES-19
GDI	*QGDI-PA × P.ipbb-1D.2*	1D	64.4–88.0	9.2	19	4.76	Pamyati Azieva	KRIAPG-17, KRIAPG-18, KRIAPG 4-years mean; NKAES 17, NKAES 3-years mean
GDI	*QGDI-PA × P.ipbb-2B*	2B	0.0–13.3	3.3	5	−2.6	Paragon	KRIAPG-17; NKAES-17
GDI	*QGDI-PA × P.ipbb-4B*	4B	33.1–59.6	3.2	6	2.77	Pamyati Azieva	KRIAPG-17; NKAES-17
GDI	*QGDI-PA × P.ipbb-5A*	5A	63.2–87.9	3.9	7	0.42	Pamyati Azieva	KRIAPG-18; NKAES-19
SED	*QSed-PA × P_ipbb-1D*	1D	0.0–11.9	16.9	50	14.4	Pamyati Azieva	KRIAPG-16–KRIAPG-19, KRIAPG 4-years mean/NKAES-17–NKAES-19, NKAES 3-years mean
SED	*QSed-PA × P.ipbb-6B*	6B	28.4–66.0	5.8	8	−3.02	Paragon	KRIAPG-16, KRIAPG 4-years mean
SED	*QSed-PA × P.ipbb-7A*	7A	52.2–73.1	3.5	12	3.96	Pamyati Azieva	KRIAPG-17, NKAES-17

Note: TWL—grain test weight per liter (g/L); GPC—grain protein content (%); GC—gluten content (%); GDI—gluten deformation index (unit); SV—sedimentation value (mL); GSC—grain starch content (%); TKW—thousand kernel weight (g); YM2—yield per square meter (g/m^2^).

## Data Availability

The original contributions presented in this study are included in the article/Supplementary Material. Further inquiries can be directed to the corresponding author.

## Supplementary Materials

The following supporting information can be downloaded at https://www.mdpi.com/article/10.3390/plants14111728/s1, Table S1: The raw field data at the Kazakh Research Institute of Agriculture and Plant Growing (KRIAPG, Almaty region, Southeast Kazakhstan) and the North Kazakhstan Agricultural Experimental Station (NKAES, Petropavlovsk, North Kazakhstan). Table S2: List of all QTLs identified in the Pamyati Azieva × Paragon mapping population. Table S3: List of stable QTLs identified in the Pamyati Azieva × Paragon mapping population. Table S4: List of identified QTLs based on grain quality traits of the Pamyati Azieva × Paragon mapping population and comparative analyses with the associations revealed in previously published reports. Table S5: List of pleiotropic loci-associated grain quality, yield components, and adaptation traits in Pamyati Azieva × Paragon mapping population.
